# Differential Protein Network Analysis of the Immune Cell Lineage

**DOI:** 10.1155/2014/363408

**Published:** 2014-09-21

**Authors:** Trevor Clancy, Eivind Hovig

**Affiliations:** ^1^Department of Tumor Biology, Institute for Cancer Research, The Norwegian Radium Hospital, Oslo University Hospital, 0310 Oslo, Norway; ^2^Biomedical Research Group, Department of Informatics, Faculty of Mathematics and Natural Sciences, University of Oslo, 0310 Oslo, Norway; ^3^Institute of Cancer Genetics and Informatics, The Norwegian Radium Hospital, Oslo University Hospital, 0310 Oslo, Norway

## Abstract

Recently, the Immunological Genome Project (ImmGen) completed the first phase of the goal to understand the molecular circuitry underlying the immune cell lineage in mice. That milestone resulted in the creation of the most comprehensive collection of gene expression profiles in the immune cell lineage in any model organism of human disease. There is now a requisite to examine this resource using bioinformatics integration with other molecular information, with the aim of gaining deeper insights into the underlying processes that characterize this immune cell lineage. We present here a bioinformatics approach to study differential protein interaction mechanisms across the entire immune cell lineage, achieved using affinity propagation applied to a protein interaction network similarity matrix. We demonstrate that the integration of protein interaction networks with the most comprehensive database of gene expression profiles of the immune cells can be used to generate hypotheses into the underlying mechanisms governing the differentiation and the differential functional activity across the immune cell lineage. This approach may not only serve as a hypothesis engine to derive understanding of differentiation and mechanisms across the immune cell lineage, but also help identify possible immune lineage specific and common lineage mechanism in the cells protein networks.

## 1. Introduction

Recently, the Immunological Genome Project (ImmGen: http://www.immgen.org/) consortium completed the first phase of their objective to generate a comprehensive resource of the gene expression repertoire across all murine immune cells [1–5]. This massive genomics effort is the so-called “*act one*” [4] in the characterization of the molecular circuitry across immune cells. The ImmGen effort has the goal to chart the entire immune regulatory mechanisms of the hematopoietic cell lineage. Rigorous standardization procedures in flow cytometry and gene expression microarrays are applied to build this resource, resulting in a comprehensive database of the gene expression profiles in the murine immune system. This present study is motivated by the possible benefit of auxiliary bioinformatics analysis of the ImmGen resource. The aim of such additional bioinformatics examination of this rich resource is to help catalyze the process of understanding the molecular mechanisms of differentiation and functional activity across the entire spectrum of hematopoietic cells.

As is often the case with global or systems profiling of immune cells, the experimental approach to collate the data often concentrates on either protein interaction networks, using proteomics based approaches, or gene regulatory networks, using gene expression microarrays. Usually, these two key types of molecular data sources are not integrated in a combined analysis [6]. Integrated proteomics and transcriptomics analysis may elucidate the underlying differential molecular functions being driven by the regulatory networks across all immune cells. Additionally, in the few immunological studies that do incorporate a global systems approach to dissect the complex network of relationships in immune cells a limited set of hematopoietic cell lineages are studied.

In this meta-analysis, we provide an integrative bioinformatics characterization of the immune cell specific protein networks associated with the gene expression profiles from the ImmGen resource. The generation of such a protein network perspective on the ImmGen regulatory networks may offer immunologists an opportunity to derive hypotheses, which can be tested experimentally and either confirmed or refined through further* in silico* and experimental analyses.

The bioinformatics integration of protein networks with large gene expression datasets was demonstrated, over a decade ago, to be highly useful in the elucidation of signaling functions [7]. Bioinformatics algorithms have continued to successfully demonstrate the ability to identify key functional relationships through challenging task of integrating transcriptomics and interactome datasets [8, 9]. Such network integration of data has since often proven to be effective in generating hypotheses to study the precise mechanisms in signaling networks which correspond to the observed changes in the gene expression profiles. One example of this approach is the integration of protein interaction networks and gene expression data using tissue specific gene expression profiles, which has proven to be insightful in recent years in predicting aspects of tissue specific cell biology [10]. Similarly, in this study we perform a tissue specific protein network analysis based exclusively on the immune cell lineage. To that end, we employ the resources at the Immunological Genome Project, which consists of 816 gene-expression profiles across the mouse immune cell lineage and has recently been systematically analyzed to generate the gene regulatory circuits for each of the cell types in the immune cell lineage. This latter effort, algorithmically named as OntoGeNet, for the first time allowed for the comprehensive identification of their potential regulatory modules [11]. In this study, we use affinity propagation to describe a protein network perspective of these gene regulatory networks driving the gene expression across the immune cell lineage through integrative bioinformatics approach to compute differential protein networks functions. Affinity propagation was applied to a protein interaction network similarity matrix to integrate the gene targets of the immune cell regulatory network with possible interaction network mechanisms. This bioinformatics approach can be applied to generate insights into the underlying mechanisms governing the differentiation and the differential functional activity across the entire murine immune cell lineage.

## 2. Material and Methods

### 2.1. Sources of Mouse Protein Networks

Protein interactions were sourced from 10 integrated protein interaction databases, as organized by the iRefIndex [12], and were extracted from the binary physical protein interactions through download from iRefWeb [13, 14]. The protein interactions, their annotations, and their identifiers are integrated in this resource using the iRefIndex method by mapping protein identifications across the databases, enabling systematic backtracking to establish the nonredundant identity of the interaction partners. A strict filtering process for each protein interaction was applied, whereby we selected only physical binary protein interactions from the iRefWeb that satisfied all of the following criteria: (a) experimentally verified; (b) within the same organism; (c) at least one supporting publication in Medline, and (d) physically binding protein interactions. This resulted in a mouse protein interaction network consisting of 20,200 binary protein interactions.

### 2.2. Integration of Immune Lineage Regulatory Modules with the Murine Protein Interactome

For the immune-cell lineage specific information, we utilized the ImmGen consortium data set (April 2012 release) which consisted of 816 expression profiles from 246 cell types of the mouse immune system [1]. We used each of the 7965 ImmGen genes assigned to a network module from the OntoGeNet algorithm, as a source to generate a protein network similarity matrix (PNSM) from a pairwise analysis of these genes (described below). Each target of all 81 course regulatory network modules calculated from the OntoGeNet algorithm [11] on the ImmGen resource was integrated into a bipartite network analysis as described below. Of the 7965 genes among the ImmGen modules, 2133 had at least one network partner in the mouse interactome, reflecting the current incomplete state of interactome databases [15]. The range of coverage of each individual module in the interactome is illustrated in Supplementary Figure 1 in the Supplementary Material available online at http://dx.doi.org/10.1155/2014/363408, which plots the percentage of genes in each module holding at least one interaction in the mouse interactome (where 2 modules had zero interactors and many have greater than 25% coverage within the module, with a maximum of 48%).

The cell types analyzed encompass all the main hematopoietic lineages, including stem and progenitor cells, granulocytes, monocytes, macrophages, dendritic cells (DCs), natural killer (NK) cells, B cells, and T cells. The T cells include many important subtypes types of α*β* T cells, regulatory T cells (T_reg_ cells), natural killer T cells (NKT cells), and *γ* *δ* T cells. A table of the known global regulators that modulate the gene expression programs of these immune cell lineages, as used in this study, is illustrated in Table 1. These regulators inform the selection of target genes selected based on application of OntoGeNet on the known regulators of the immune cell lineages in mice. OntoGeNet is an algorithm recently developed to achieve the reverse engineering of lineage-specific gene regulatory modules from the ImmGen gene-expression profiles. It has an innovative feature in that it integrates the lineage tree when predicting its gene regulatory networks, in addition to the gene expression activity, such that the module's genes are recapitulated in related cell types.

### 2.3. Defining a Measure of Protein Interactome Similarity Scores to Generate Protein Network Similarity Matrices

A protein network similarity matrix (PNSM) was built and mapped to the ontogeny of the murine immune cells gene expression profile as catalogued at ImmGen. Each of the 7965 target genes from the gene regulatory network modules in OntoGeNet was mapped to its protein product counterparts in the mouse protein interaction network (of which 2133 had at least one interaction). We calculated a protein interaction similarity index for all these gene pairs, which compose the PNSM. The pairwise calculation of the protein interaction network similarity scores across all genes in the murine protein networks was calculated by using the Simpson similarity score. For any given pair of genes,* A* and* B*, their shared interaction partners were calculated as (*N*(*A*)∩*N*(*B*)) in relation to the degree of *N*(*A*) and *N*(*B*) of the mouse protein interaction network overall. The Simpson index was then calculated as the proportion of shared protein interactions between the gene pair relative to the degree of the least-connected gene in the murine protein interaction network.

(1) $$\begin{array}{c} \frac{N\left(A\right)\cap N\left(B\right)}{\min\left(\left|N\left(A\right)\right|,\left|N\left(B\right)\right|\right)}. \end{array}$$

For each of these similarity scores, a real-valued matrix* S* was constructed, in which an entry *S*_*AB*_ corresponded to a value measuring how similar gene *A* was to gene *B* in the mouse interactome.

The Simpson index captures the proportion of shared interaction partners between each gene pair, relative to the degree of the least-connected gene [16]. The choice of using Simpson index as the similarity score to build the PNSM was motivated by its effective comparison of two diverse sets of gene pairs in the network, and by so doing not penalize pairs which have large differences in their node degree in the interactome network [16]. Such large differences often occur in molecular networks due to the scale free property of these networks, as well established in recent years [17]. Other similarity indices can be used to compare networks, each having their strengths and weaknesses depending on the biological application. The Jaccard index, for example, effectively captures the proportion of shared nodes between the gene pairs. However this is in proportion to the total number of nodes in both genes irrespective of their individual node degree. Other similarity indexes are more suitable to capture communities within networks or predict biological function, and all of these indices have been extensively surveyed recently [16] and their optimal use characterized.

### 2.4. Affinity Propagation Applied to the Protein Interaction Similarity Matrix (PNSM) to Identify Exemplar Protein Network Signatures

In recent years there has been a great deal of development of methods to detect clusters, modules, or communities in molecular networks [18, 19] and also to predict the interrelatedness of these groups [20, 21]. The strengths and weaknesses of these different methods have been profiled in recent bioinformatics perspectives [22]. In this study affinity propagation (AP) was applied to the PNSM to identify the differential protein interaction network mechanisms associated with each of the 10 immune cell types analyzed [23, 24]. Affinity propagation holds an advantage over other clustering procedures applied to similarity matrices in that the method does not require values to be in a specific range. Additionally, although there are similar methods which compete with the performance of AP applied to smaller networks [25], the AP method performs optimally on large similarity matrices [26] such as the PNSM developed in this study whereby all pairwise datapoints are considered as candidate exemplars (clusters). Additionally, the exact number of clusters does not need to be specified. Furthermore, the choice of using the AP algorithm for clustering the similarity matrices has the advantage over classical clustering methods in the fact that AP can determine the appropriate number *k* of clusters, depending on a vector of the median of similarities as input preference for all genes in the mouse genome. The suitability of AP applied to the PNSM for the functional analysis of the immune cell lineage in this study may also be delineated by the feature of AP not only to identify clusters but also to capture compressed information summarizing the identified clusters [23, 27].

The affinity propagation procedure analyzed measures of protein interaction network similarity between all pairs of proteins built into the PNSM and simultaneously considered all pairwise comparisons as potential exemplars (exemplars, in this setting, are groups of proteins which have similar interactions partners in the mouse interactome). Real-valued matrix so-called “messages” which passed between each pairwise comparison are exchanged between data points until a high-quality set of exemplars (or clusters) eventually emerge as the algorithm iterates. In affinity propagation, there are two different types of messages exchanged between each pairwise similarity in the PNSM: “availabilities” (*a* (*A*, *B*)) and “responsibilities” (*r* (*A*, *B*)). The “availability,” sent from candidate exemplar gene* B* to data point gene* A*, is a reflection of how each gene* B* is suitable to be available for gene* A* to become an exemplar cluster. The “responsibility,” sent from data point gene* A* to candidate exemplar gene* B*, is a reflection of how each gene* A* is suitable to serve as exemplar in* B*. The PNSM, a real-valued matrix *S*_*AB*_,   *s* (*A*, *B*) described above, is taken as input for the affinity propagation algorithm. Each data point is assessed for its suitability to be a candidate exemplar. The details of the updating functions computed as affinity propagation iterates are described extensively by Frey and Dueck [23]. Briefly, the availabilities and responsibility functions are computed as log likelihood ratios, reflecting the evidence accumulated iteratively for how well suited each data point may serve as a candidate exemplar. Initially, the availability is set to zero. The responsibility updates are then computed as

(2) $$\begin{array}{c} r\left(A,B\right)⟵s\left(A,B\right)-\max\left\{a\left(A,B^{\prime }\right)+s\left(A,B^{\prime }\right)\right\}. \end{array}$$

The above function allows all candidate exemplars in the PNSM to compete for inclusion of a data point. The availability update then accumulates evidence scores from all data points as to whether each candidate exemplar has likelihood of emerging as an optimal exemplar, using the following update function:

(3) $$\begin{array}{c} a\left(A,B\right)⟵\min\left\{0,r\left(B,B\right)+\sum \max\left\{0,r\left(A^{\prime },B\right)\right\}\right\}. \end{array}$$

This availability update functions sets the availability of a candidate exemplar to the sum of the positive responsibilities, *r* (*A*, *B*); the candidate exemplar receives from all other data points plus the self-responsibility *r* (*A*, *B*). This self-responsibility, *r* (*B*, *B*), is an evidence score that ranks whether gene* B* is an exemplar based on the input preferences in the procedure. More extensive details of the affinity propagation algorithm are available in the Frey and Dueck paper [23], which described its development. The functional analysis on the computed exemplars was performed based on structured vocabularies from the Gene Ontology project [28] biological process tree, using a combination of DAVID functional association tools [29] and the gene set enrichment analysis, through the use of GSAT [30, 31]. The bipartite networks, which illustrate the properties of the computed protein networks exemplars with activators of immune cell differentiation, the groups (modules) of coexpressed genes from ImmGen, and the immune cell types, were visualized using the Cytoscape network analysis toolkit [32].

## 3. Results and Discussion 

### 3.1. Affinity Propagation on Protein Network Similarities, Informed by the Immune Cell Lineage

**Figure 1:**
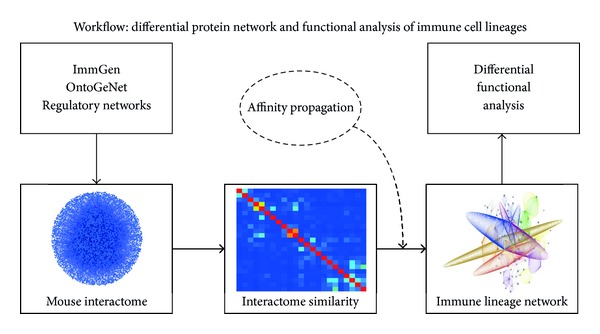
Workflow of affinity propagation on the PNSM and the differential network analysis. The known activator genes which drive the differentiation of the main immune cell types (see Table 1) were used to query the list of their target genes as computed from the OntoGeNet algorithm on the ImmGen resource. The protein network neighborhood of each of the 7965 genes assigned to an ImmGen network module of was integrated with their target lineage information, computed from an integrated set of validated protein interaction network databases. Then, using the Simpson similarity index, their PNSM was computed. Affinity propagation or “message passaging” was then applied on the PNSM, to capture features of the immune lineage network. The resulting exemplars computed from the affinity propagation allows for differential functions to be captured through the lineage tree.

**Figure 2:**
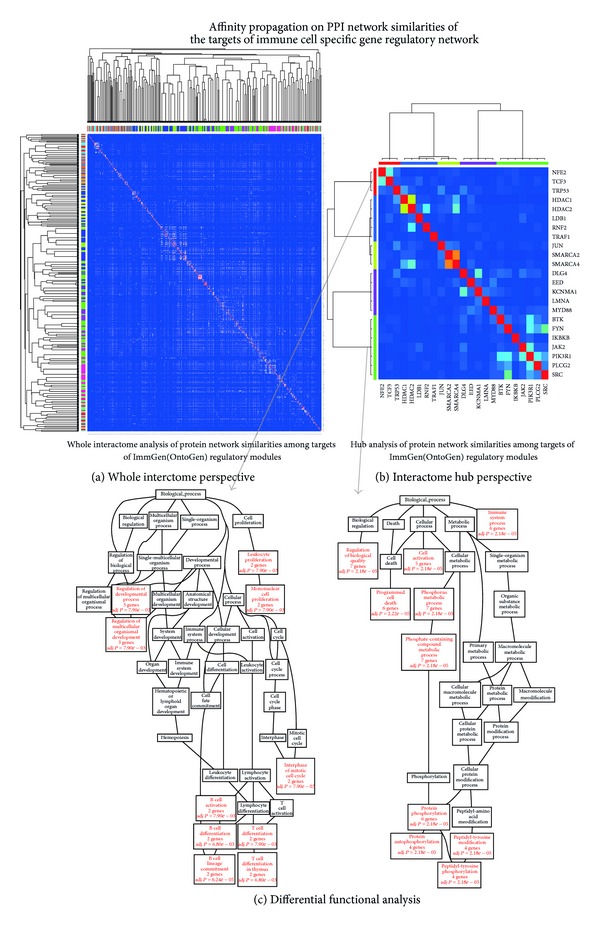
Affinity propagation clustering of the protein interaction association similarity matrix of OntoGeNet target module genes. (a) The PNSM is illustrated for both the entire list of target genes computed from the OntoGeNet algorithm on the ImmGen resources. The degree of red color in the heatmap corresponds with the strength of similarity in the protein network for each gene pair. The exemplars as computed from affinity propagation are illustrated in the annotated color bars and the resulting hierarchical clustering (see Table 1 for list of the protein network exemplars). (b) Exemplifies the effect of application of the affinity propagation workflow applied to protein interaction networks, on hubs only. The hub analysis highlights the possible most influential interaction mechanisms activated by the gene regulatory networks (OntoGenet), which govern the immune cell lineage. (c) A differential functional analysis using the Gene Ontology Biological Process (GO-BP) tree is illustrated for two of the computed exemplars. The trajectory of functional significance of GO-PB terms from the two exemplar's genes from Figure 2(b) (indicated by the arrows) is illustrated through the GO-BP tree. GO-BP terms significant for the gene list within the exemplars are highlighted in a red color.

A protein interaction network similarity matrix (PNSM) was built by computing the similarity in protein interaction partners for all pairwise combinations of genes assigned to modules in ImmGen having been identified as targets of the transcriptional activators driving immune cell lineages as computed from the OntoGeNet algorithm. Affinity propagation was applied on the PNSM in order to identify groups of genes (or “exemplar” clusters), which correspond to similarity in protein interaction network partners. The outcome of this particular approach is the computation of exemplars (or clusters) of genes that share similar protein interaction partners in the mouse interactome. Integrating this level information with the gene regulatory network information captured by OntoGeNet [11] using the ImmGen resource allowed for the capturing of common functional mechanisms among the immune cell lineage. The workflow of the approach applied to achieve this bioinformatics integration of diverse datasets in systems immunology is described in Figure 1. The outcome of the workflow defined by this strategy is illustrated in Figure 2(a). The resulting exemplars (characteristic groups of proteins which have similar interaction partners in the mouse interactome) computed by the affinity propagation are both illustrated in Figure 2 and listed in Supplementary Table 1. It is evident that exemplars with diverse functions were captured using the approach. The nature of this diversity was also illustrated using the same workflow implemented on target proteins that are also hubs in the mouse protein interaction network (as depicted in Figure 2(b)). A differential functional analysis using the Gene Ontology Biological Process (GO-BP) tree was also applied using gene set enrichment statistical approaches to analyze some of the resulting exemplars [31]. This differential functional analysis is illustrated for two of the computed exemplars in Figure 2(c). The trajectory of functional significance of GO-PB terms from selected two exemplar's genes in Figure 2(c) highlights immune relevant yet diverse, functional mechanisms captured and possibly implicated as important, in immune lineage differentiation. The two GO-BP functional analyses illustrated in Figure 2(c) highlight significant associations for immune relevant terms, and the different exemplars protein networks which were used to generate these associations have differential functional paths through the GO-BP process tree.

### 3.2. Diverse Quantity and Type of Protein Network Exemplars Associated to Gene Expression Programs among the Immune Cell Lineage

**Figure 3:**
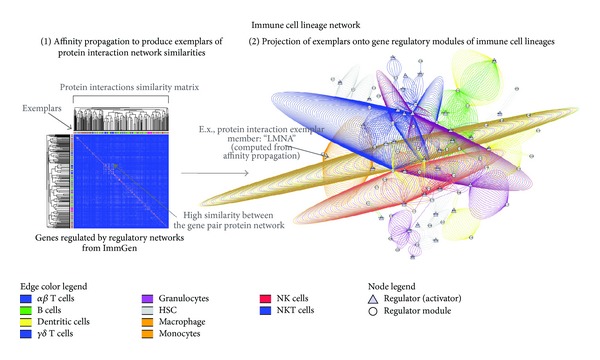
Integrated protein interaction networks perspective on the gene regulation networks driving immune cell lineages. The immune cell lineage network is depicted as a bipartite network with multiple edges representation. Each edge represents a protein network exemplar. The multiple edges connect the different node types and reflect the regulator activity superimposed on the multiple protein network exemplars activated by immune lineage regulators. Each relationship is a representation of the gene regulation modules from the ImmGen resource connecting with the known regulators of immune cell lineages. Each edge in the network represents a relationship between an immune cell line lineage type (see legend in Figure 3(1)) and one of the known activating factors regulating the differentiation of that lineage (see Table 1 for list of the known activators used). An edge is drawn in the network if there is connection between a regulator gene (triangle node) and a course module (groups of commonly expressed genes) calculated from OntoGenet (circle nodes). The number of lines between a regulator and a module is a measure of how many “protein network exemplars,” as calculated from the affinity propagation (see main text), are associated to the regulatory module (and therefore a possible measure of the diversity of signaling networks activated in driving the lineage of the immune cell type).

In Figure 3, a bipartite representation of the affinity propagation on the PNSM is depicted. This bipartite network of protein network exemplars and OntoGeNet gene expression modules driven by well-known activators of immune lineage gene expression (Transcription factors from Table 1) is illustrated using multiple edges, whereby each edge is color coded to represent an exemplar as computed using the affinity propagation algorithm on the PNSM. This immune lineage network in Figure 3 illustrates how certain specific clusters in the protein similarity network (exemplars) are potentially activated in specific immune cell types, as indicated by the single color of the respective edges representing the protein network exemplars, while others are potentially activated in multiple lineages during immune cell differentiation, as indicated by many colors of the respective local network. From the strategy employed in this study, as outlined in Figure 1, it is apparent, for example, that there are possibly many distinct protein interaction network mechanisms (as represented by the multiple exemplar edges) associated in the bipartite network with the T cell lineage (where the protein network exemplars are color coded as blue edges in the network), some of which are also shared with natural killer (NK) cells (where the protein network exemplars are color coded as red edges in the network). This NK cell and T cell example are indicative of shared protein network functions in these two different immune cell lineage types, which may be implicated in their shared cytotoxic abilities and immune cell effector functions. The observation is interesting when considering that although NK cells are not generated in the thymus, they share some key molecular characteristics and protein interactions with T cells. For example, they both have some common surface markers. Additionally, NK cells also use the same generic killing mechanisms as cytotoxic CD8^+^ T cells, although NK cells do not have rearranged T-cell receptor molecules and therefore belong to the innate immune system. Although being regulated by different activators, most of the immune cell types appear to be activated by some common mechanisms with the cells protein network (as illustrated by the activator to module relationships hosting protein network exemplars associated to multiple colors representing all the immune cell types).

Additionally, the immune cell lineage network in Figure 3 also illustrates protein network exemplar relationships that possibly conform to immune cell lineage specific functions (as illustrated by the activator to module relationships hosting protein network exemplars associated to single color representing the immune cell type). This is exemplified in the exemplars specific to the hematopoietic stem cell (HSC) lineage depicted as grey edges in the bipartite network in Figure 3. Here, the protein network exemplars activated in the HSC immune cell types are not activated in others in the lineage network, indicating a possible diminishing importance or deactivation of these functions as immune cells terminally differentiate beyond the HSC lineage.

### 3.3. Functional Similarity from a Lineage Specific and Common Lineage Perspectives

**Figure 4:**
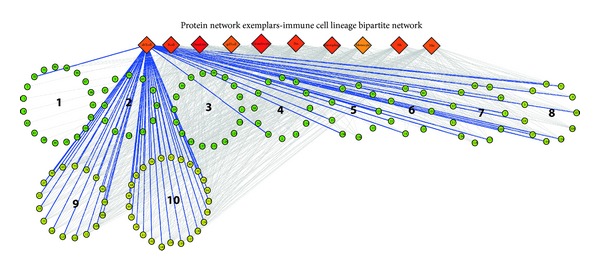
Bipartite network representation of immune cell lineages and protein network exemplars. In this bipartite network representation, the protein network exemplars are represented as circles (the names and genes in these exemplar groups are listed in Table 1), and immune cell types represented as square diamonds. A color gradient of degree of the node in the bipartite network ranges from green (lowest) to yellow (intermediate) and red (highest) is represented. Lineage specific exemplars are clearly illustrated in addition to the increasing range of common protein network exemplars. (These two types of patterns are listed in Table 2 and 3, resp.). The protein network exemplars are ordered, 1−10, according to their connectivity to the ten immune cell types in the bipartite network.

A different node-type of bipartite network representation of the protein network exemplars with the ten different immune cell types are represented in Figure 4. In this network, the two nodes categories are immune cell types and the calculated protein network exemplars. It is evident that the strategy of applying affinity propagation on the PNSM allows for the capturing of both lineage specific and shared protein network exemplars with diverse functions implicated. These clusters correspond to groups of proteins whose interactions in the cell are possibly more important for the functional activity of lineage specific and common immune cell lineage functions. Such bipartite network representations of protein network exemplars and immune cell types may serve as useful descriptions of both common and more specialized protein interaction functions among immune cell types. In Table 2, an overview of the range of lineage specific protein network exemplars illustrated in the bipartite network in Figure 4 is provided, with some indication of their functional associations. Similarly, lists of those protein network exemplars identified as common to all ten immune cell types analyzed are provided in Table 3.

The gene functions associated with the lineage specific exemplars range from a possible activation of a cell specific Jak-Stat pathway in hematopoietic stem cells (HSC) to the lineage specific gene regulation activity in dendritic cells (see Table 2). It is interesting to note that the Jak-Stat pathway is established to be critically important in regulation of the differentiation mechanisms among stem cells. Additionally, mutations in the Jak-Stat pathway are known to cause destabilization of HSC homeostasis and lead to many blood disorders [33]. Notably, many of the lineage specific exemplars in supplementary Table 1 and the bipartite network in Figure 4 are associated to HSCs (42%). This is also illustrated in the network of regulatory and exemplar activity in Figure 3. The increasing degree of promiscuity among the protein network exemplars is evident in the bipartite network by the number of connections of an exemplar to different immune cell type ranges from lineage specific associations to increasingly common lineage associations. One such common lineage protein network exemplar listed in Table 3 is cytokine-cytokine receptor signaling, centered on tumor necrosis factor (TNF) superfamily protein interactions (associated to all ten immune cell types in the bipartite network). As expected, such important protein interactions are preserved across all ten of the protein lineages identified, as TNF-TNF receptor signaling mechanisms are critical for the intercellular communication common to immune cell activity ranging from cell proliferation to apoptosis of immune cell populations.

## 4. Conclusions 

In this study, we have described how the integration of protein interaction networks with the most comprehensive database of gene expression profiles of the immune cell lineage (ImmGen) can be used to generate insights into the underlying mechanisms governing the differentiation and the differential functional activity across all immune cell types. To perform this bioinformatics integration efficiently, and at a large scale, we used affinity propagation applied on a similarity matrix of immune lineage targets gene's interaction partners in the mouse interactome (the PNSM). The approach outline here not only may serve as hypothesis engine to derive understanding of differentiation and mechanisms across the immune cell lineage, but also help identify possible immune lineage specific and common lineage mechanism with the protein networks of the various cell types.

The potential value in applying affinity propagation on the mouse PNSM as a viable strategy to characterize protein network clusters important for immune cell function in human studies is a questionable issue, considering the evolutionary distance between mouse and human [34]. It could be argued, therefore, that bioinformatics strategies such as that described in this study may not be directly applicable to human studies which attempt to capture signatures of immune cell activity using protein networks [35]. However, as mouse is an often used and a powerful model organism for human medicine, it will be exciting to assess the impact of this and similar bioinformatics procedures on the inference of activated protein networks exemplars in disease associated hematopoietic cells in mice models. One such powerful application, for example, is that of gene expression profiles tumor specific CD4^+^ T cells in a mouse model of multiple myeloma [36], which could possibly capture tumor specific protein interaction network mechanism using the approach described here. Additionally, the gene regulatory network programs we used from OntoGeNet and the expression profiles from ImmGen are conserved between mice and human [37]. With that in mind it is likely that many inferences can be made from mouse model to human immune cell biology using the bioinformatics strategy described here. A natural extension, however, is to apply this strategy to infer protein interaction network mechanisms on similar projects to ImmGen from the compendiums of human immune cells. Namely, the Human Immunology Project Consortium (HIPC) is currently developing standards for data collection, integration, data exchange, and development of a central database of systems immunology data in human samples. The bioinformatics approach described may be fruitful when applied to these and similar upcoming large-scale data sets. Such a protein network integration of the immune system's gene expression compendiums of model organisms may help identify protein interaction mechanisms which are shared among immune types linked to their differentiation, in addition to immune lineage specific immunological mechanisms in protein networks.

## Supplementary Material

Supplementary table one is a listing of all computed exemplars and a listing of the genes within each exemplar. Supplementary Figure 1 illustrates the coverage each ImmGen module has in the mouse protein interaction network.

## Figures and Tables

**Table 1:** Known transcriptional activators of the immune cell lineages.

Immune cell type	Known transcriptional activators
B cells	POU2AF1, PAX5, EBF1, SPIB, SFPI1, FOXP1
Dendritic cells	RELB, CIITA, AHR, SPIB, SFPI1
Granulocytes	CEBPB, NFE2, SFPI1, FOXO3, CEBPE, FLI1
Hematopoietic stem cells	HLF, LMO2, MYC, MYCN, GATA2, MEIS1, E2F6
Macrophage	CEBPA, CEBPB, SFPI1
Monocytes	CEBPB, SFPI1
Natural killer cells	EOMES, TBX21, SMAD3, GATA3
Natural killer t cells	GATA3, ZBTB16
abT cells	TCF7, BCL11B, GATA3, IKZF2, RORC, SMAD7, TOX
gdT Cells	GATA3, SOX13, ID3

**Table 2:** Lineage specific protein network exemplars.

Exemplar ID	Genes assigned to the protein network exemplar	Functional annotation	*P* value	Immune cell type
102	EPOR, PTPN1, STAT5B	Jak-STAT signaling pathway (KEGG)	5.20*E* − 02	Hematopoetic stem cells

20	CCNT1, EIF2B1, MYC			Hematopoetic stem cells

86	EZR, NGFRAP1, NTF3	Neurotrophin signaling pathway	4.50*E* − 02	Granylocytes

49	GRB7, TIA1			Hematopoetic stem cells

104	ARRB1 BGN PTS			Macrophages

96	PLCB2, POLA1, VIM			Granylocytes

98	POT1A, TERF1	Telomere maintenance via telomerase (GO-BP)	5.90*E* − 04	Hematopoetic stem cells

7	AR, ATRX, CTCF, SMC1A, SMC3	Cell cycle (KEGG)	4.40*E* − 02	Dendritic cells

119	CRE, PRPF40A, SMC2	Nucleoplasm (GO-CC)	8.00*E* − 02	Dendritic cells

61	CLDN11, CNOT6L, ITGA5, ITGB1, SPARC	Cell adhesion molecules (CAMs) (KEGG)	7.80*E* − 02	Macrophage

10	BICC1, CREBBP, CSK, KHDRBS1, PRMT1, RBM39	Control of Gene Expression by Vitamin D Receptor (KEGG)	7.00*E* − 02	Ggranylocyte

97	EIF3L, POLR1B, POLR1E	RNA polymerase (KEGG)	4.70*E* − 03	Hematopoetic stem cells

41	BRCA2, CEBPD, FANCD2	Cell cycle process (GO-BP)	5.70*E* − 02	Macrophages

70	ADRB2, DLL1, MAGI3	Plasma membrane (GO-CC)	5.40*E* − 02	abTcells

88	CHORDC1, IGBP1, NR3C1, PPP5C	Transition metal Ion binding (GO-MF)	3.90*E* − 02	Hematopoetic stem cells

75	MEOX1, MEOX2, TLE4	Transcription factor activity	3.40*E* − 03	Granylocytes

42	FBXW7, NOTCH1, NPM1 STAT4	Notch signaling pathway	1.20*E* − 02	Hematopoetic stem cells

36	AEBP2, EED, MORC3, SETX, UHRF1	Nucleoplasm (GO-CC)	2.30*E* − 03	Dendritic cells

38	EIF3A, EIF3B, EIF3I, EIF4E	Translational initiation (GO-BP)	2.00*E* − 08	Hematopoetic stem cells

**Table 3:** Common protein network exemplars.

Exemplar ID	Genes assigned to the protein network exemplar	Functional annotation	*P* value
127	TANK, TNFRSF11A, TNFRSF4, TNFRSF9, TRAFD1, ZBP1	Cytokine-cytokine receptor interaction (KEGG)	3.50*E* − 06

32	ATP6V1A, BAIAP2, CAMK2D, CLU, DLG4, DYNLL1, EPS8, FZD1, FZD4, GRIA3, HCK, INADL, KCNJ10, NSF, OPRD1, PACSIN1, PACSIN2, PGK1, PHB2, PPP3CA, RGS12, SEMA4B, SEMA4C, SLC9A3R1, STXBP1, SYT1	Wnt signaling pathway (KEGG)	1.60*E* − 03

44	CD2AP, DAPK1, EFNA2, EPHA4, FGR, FYN, PKD2, RAVER1, RGS1, SH2D1A, SH3BP1, SLAMF1, VAV1, VAV2, ZAP70	T cell receptor signaling pathway (KEGG)	26.7

93	HNRNPA1, LGALS3, NCOA3, PIAS1, RNF19A	Transcription cofactor activity (GO_BP)	5.30*E* − 02

29	CLNK, FYB, LYN, PECAM1, SKAP2	Leukocyte activation (GO_BP)	6.30*E* − 02

62	CSF2RB, EIF2AK2, GHR, GMCL1, GNB2L1, HES1, IFNGR1, IL6ST, JAK1, JAK2, JAK3, LMO4, NCL, PAG1, SLC40A1	Jak-STAT signaling pathway (KEGG)	2.10*E* − 09

139	BRCA1, CIITA, GTF2H2, KDM5D, PDLIM4, POLR1A, POLR2B, TRIP4, TRPS1, ZFP111, ZFP292	Zinc ion binding (GO_BP)	5.30*E* − 07

135	RIPK2, TAX1BP1, TIFA, TNFAIP3, TNIP2	Apoptosis (GO_BP)	6.70*E* − 03

112	ANXA2, BMYC, GAB2, PRKCE, PRMT5, S100A10	Fc epsilon RI signaling pathway (KEGG)	1.40*E* − 02

140	CCNG1, CCNG2, E2F1, GSTA4, NR4A3, SWAP70, TRIM32, TRP53	Cell cycle (GO_BP)	1.60*E* − 03

124	HSPA5, HSPA9, HSPD1, PDXK, RAPGEF4, STIP1, YWHAE	Adenyl ribonucleotide binding (GO_MF)	6.60*E* − 04

79	ALDOA, CD40, IL1R1, IL1RAP, IL1RL1, IRAK3, IRAK4, IRF4, IRF5, LRRFIP1, MYD88, TIRAP, TLR4, TNFRSF13B, TUBA1A	Cytokine-mediated signaling pathway (GO_BP)	2.50*E* − 07

126	BCAR1, BLK, CD22, CD247, CLEC7A, ERBB2, IL15RA, ITGB3, RANBP2, SYK, TUBA4A, WIPF1	Cell surface receptor linked signal transduction (GO_BP)	4.10*E* − 03

21	CCL3, CCL4, CCR1, CCR3, CCR5	Chemokine signaling pathway (GO_BP)	9.80*E* − 07

63	APOE, CASK, CNN3, CTTN, HSP90B1, IPO11, KCNMA1, LDHA, MYO5A, NDEL1, NUDC, PRDX2, RAB6B, ROCK2, SH3BP4, TPM1, TPT1, TRF, TUBB5	Cellular homeostasis (GO_BP)	9.20*E* − 04

80	MYO1C, PCDH15, RICTOR, RRN3, VPS35	Cytoskeleton organization (GO_BP)	9.30*E* − 02

92	GFR, PDGFRA, PDGFRB, PTEN, SLC9A3R2	Transmembrane receptor protein tyrosine kinase signaling pathway (GO_BP)	2.80*E* − 06

142	CSNK1E, NXN, PLCG2, RAD51, VANGL1, VANGL2	Wnt signaling pathway (KEGG)	3.90*E* − 03

48	GRB10, IGF1R, MAP3K5	Insulin-like growth factor receptor signaling pathway (KEGG)	1.30*E* − 03

50	HCST, IL2RB, KLRK1, TYROBP	Integral to membrane (GO_BP)	9.50*E* − 02

52	ANP32A, DACH1, FOSB, HDAC2, HDAC9, L3MBTL2, MTA1, MTA3, RBBP7, REST, SP3, TCF7L2, WDR5, ZDHHC13, ZFPM1	Regulation of transcription (GO_BP)	4.00*E* − 09

116	F2RL2, MAP3K2, SMAD1, SMAD5, TOB1, ZEB2	Cell surface receptor linked signal transduction (GO_BP)	4.60*E* − 01

59	CASP1, CASP3, CASP8, CEBPB, GZMB, IL1B	Regulation of apoptosis (GO_BP)	1.30*E* − 05

46	E2F2, GATA1, GATA3, GFI1B, LMO2, NFYA	Transcription (GO_BP)	1.30*E* − 03

94	AXL, CD19, EPHA2, IL4RA, INSR, IRS2, KRAS, NEDD9, NME2, PDCD4, PIK3AP1, PIK3CA, PIK3CB, PIK3CD, PIK3R1, PLCG1, PTK2B, RALGDS, RASSF5, SIRPA, SOCS6, TEK, TLR2	Cell surface receptor linked signal transduction (GO_BP)	2.10*E* − 03
