# Supplementary material for: Differential Protein Network Analysis of the Immune Cell Lineage
Source: Biomed Res Int. 2014 Sep 21;2014:363408. doi: 10.1155/2014/363408 (PMC4189771; doi:10.1155/2014/363408)
Supplement: Supplementary file 1 — Supplementary table one is a listing of all computed exemplars and a listing of the genes within each exemplar. Supplementary Figure 1 illustrates the coverage each ImmGen module has in the mouse protein interaction network. [file 363408.f1.pdf]

Supplementary Table 1: resulting exemplars computed by the affinity propagation

Exemplar ID	total shared pr	avg # shared pr	# genes in the e	Genes assigned to the protein network exemplar
72	802	57.286	14	AICDA,CDC6,CDC7,CDT1,DBF4,MCM2,MCM3,MCM4,MCM5,MCM6,MCM7,RPA1,RPA2,RPA3
138	252	36	7	EZH2,JARID2,MTF2,RBBP4,STK38,SUZ12,TRIM35
1	186	31	6	ANKRD32,LDB1,LDB2,RAI14,SSBP2,SSBP3
118	760	30.4	25	ACTL6A,ADD1,ARID1A,BRWD1,E2F4,E2F5,ESR1,ETS2,EYA1,GATA6,HCFC1,MED30,MYBBP1A,OGT,PBRM1,RBPJ,RIF1,SMARCA2,SMARCA4,SMARCB1,SMARCC1,SMARCD1,YEATS4,ZFP281,ZMYM2
52	316	21.067	15	ANP32A,DACH1,FOSB,HDAC2,HDAC9,L3MBTL2,MTA1,MTA3,RBBP7,REST,SP3,TCF7L2,WDR5,ZDHHC13,ZFPM1
133	126	21	6	ARNTL,CLOCK,CRY1,GTF2A1,PER1,TIMELESS
82	170	18.889	9	AP1B1,CBL,CBLB,CRK,INPP5D,NCKIPSD,PRKCA,PTPN6,USP8
108	356	16.952	21	BCOR,CBX2,CUL7,DNMT3B,E2F6,ERG,HELLS,HIST3H2A,KDM2B,LCP1,MATR3,MGA,MSH2,PCGF2,PHC3,RNF2,SCMH1,TFDP1,XRCC5,YAF2,ZBTB33
31	144	16	9	CDH1,CDH2,CTNND1,CTNND2,FHL2,IRS1,JUP,KIFAP3,RASGRF2
93	78	15.6	5	HNRNPA1,LGALS3,NCOA3,PIAS1,RNF19A
79	234	15.6	15	ALDOA,CD40,IL1R1,IL1RAP,IL1RL1,IRAK3,IRAK4,IRF4,IRF5,LRRFIP1,MYD88,TIRAP,TLR4,TNFRSF13B,TUBA1A
132	138	15.333	9	ACVRL1,CDH5,ENG,HTRA1,OCLN,TGFB1,TGFBR1,TGFBR2,XIAP
19	106	15.143	7	CCNA2,CCND1,CCNE1,CDK2,CDKN1B,HIST1H1A,NASP
91	54	13.5	4	PBX1,PBX3,POU2F1,TSC22D3
45	114	12.667	9	CCNB1,CDC25C,CDKN1A,GADD45A,GADD45B,GADD45G,PCNA,PPP1CC,REV1
134	88	12.571	7	CLDN1,CTNNA1,FUS,GJA1,MPDZ,TJP1,TJP2
21	60	12	5	CCL3,CCL4,CCR1,CCR3,CCR5
33	94	11.75	8	CD3E,DOK1,DOK2,MAP4K1,MAP4K4,NCK2,RASA1,RET
18	82	11.714	7	CBX1,CBX5,CBX7,CHAF1A,DIAP2,LBR,RBL2
17	82	11.714	7	ABL1,CABLES1,CABLES2,FMN1,GRAP2,NCK1,PXN
127	70	11.667	6	TANK,TNFRSF11A,TNFRSF4,TNFRSF9,TRAFD1,ZBP1
87	70	11.667	6	ELN,FBLN2,HSPG2,LAMC1,NID1,NID2
117	92	11.5	8	ERBB2IP,LOXL1,MAP3K1,NOTCH4,SMAD3,SMAD7,VDR,ZMYM5
85	68	11.333	6	BTRC,NFE2L2,NFKBIA,NFKBIB,NFKBIE,NR4A2
94	260	11.304	23	AXL,CD19,EPHA2,IL4RA,INSR,IRS2,KRAS,NEDD9,NME2,PDCD4,PIK3AP1,PIK3CA,PIK3CB,PIK3CD,PIK3R1,PLCG1,PTK2B,RALGDS,RASSF5,SIRPA,SOCS6,TEK,TLR2
139	124	11.273	11	BRCA1,CIITA,GTF2H2,KDM5D,PDLIM4,POLR1A,POLR2B,TRIP4,TRPS1,ZFP111,ZFP292
74	78	11.143	7	HOXA9,MEIS1,MEIS2,PKNOX1,PKNOX2,POU6F1,RNPS1
44	158	10.533	15	CD2AP,DAPK1,EFNA2,EPHA4,FGR,FYN,PKD2,RAVER1,RGS1,SH2D1A,SH3BP1,SLAMF1,VAV1,VAV2,ZAP70
9	42	10.5	4	BCL2,BCL2A1A,BCL2L1,MCL1
109	94	10.444	9	DOCK1,NCOA1,NFATC2,NRIP1,RARA,RPS6KA3,RPS6KA5,RXRA,TRIM24
29	50	10	5	CLNK,FYB,LYN,PECAM1,SKAP2
110	40	10	4	CTR9,RUVBL1,RUVBL2,WDR61
43	60	10	6	FERT2,FOS,HHEX,JUN,JUND,RARB
6	76	9.5	8	ASF1A,DHX9,HIST2H3C2,HIST2H4,MSH6,PARP1,SMARCA5,XRCC6
32	244	9.385	26	ATP6V1A,BAIAP2,CAMK2D,CLU,DLG4,DYNLL1,EPS8,FZD1,FZD4,GRIA3,HCK,INADL,KCNJ10,NSF,OPRD1,PACSIN1,PACSIN2,PGK1,PHB2,PPP3CA,RGS12,SEMA4B,SEMA4C,SLC9A3R1,STXBP1,SYT1
102	28	9.333	3	EPOR,PTPN1,STAT5B
24	46	9.2	5	CCND2,CCND3,CDK4,CDK6,NR5A2
116	54	9	6	F2RL2,MAP3K2,SMAD1,SMAD5,TOB1,ZEB2
71	44	8.8	5	IL2RG,MAPK11,MAPK14,MAPK3,MKNK1
84	44	8.8	5	NFKB1,NFKB2,NFKBIZ,RELB,RHOQ
36	44	8.8	5	AEBP2,EED,MORC3,SETX,UHRF1
128	34	8.5	4	GTF2E2,NFE2,PML,TARDBP
13	34	8.5	4	BAK1,BCL2L11,BID,BMF
38	34	8.5	4	EIF3A,EIF3B,EIF3I,EIF4E
122	58	8.286	7	ACTN1,ATG7,CYLD,IRF8,NTRK1,PRKCD,SQSTM1
62	120	8	15	CSF2RB,EIF2AK2,GHR,GMCL1,GNB2L1,HES1,IFNGR1,IL6ST,JAK1,JAK2,JAK3,LMO4,NCL,PAG1,SLC40A1
97	24	8	3	EIF3L,POLR1B,POLR1E
56	40	8	5	ID1,ID2,ID3,MED31,TCF4
16	84	7.636	11	BLNK,BTK,CMTM3,CRYAB,FLNA,H2AFZ,HAX1,LOX,MRC1,PTRF,SIGLEC1
135	38	7.6	5	RIPK2,TAX1BP1,TIFA,TNFAIP3,TNIP2
126	90	7.5	12	BCAR1,BLK,CD22,CD247,CLEC7A,ERBB2,IL15RA,ITGB3,RANBP2,SYK,TUBA4A,WIPF1
57	52	7.429	7	ARRB2,CHUK,IKBKB,IRF7,PAWR,PRKCQ,SNAP23
7	36	7.2	5	AR,ATRX,CTCF,SMC1A,SMC3
129	78	7.091	11	ATOH1,CBFA2T3,FOXP1,MLLT4,MYCN,PHF2,RUNX1T1,TAF1B,TCF12,TCF3,ZDHHC15
131	28	7	4	RUNX1,TEAD2,WWTR1,YAP1
37	28	7	4	EFNB1,EFNB2,EPHB2,SDCBP
10	42	7	6	BICC1,CREBBP,CSK,KHDRBS1,PRMT1,RBM39
142	42	7	6	CSNK1E,NXN,PLCG2,RAD51,VANGL1,VANGL2
4	28	7	4	APLP2,APP,PRNP,SIAH1A
124	48	6.857	7	HSPA5,HSPA9,HSPD1,PDXK,RAPGEF4,STIP1,YWHAE
27	34	6.8	5	CHD3,CTBP2,DNMT3A,IKZF1,IKZF3
76	34	6.8	5	FBLN1,JAG1,JAG2,MFAP5,NOTCH2
144	68	6.8	10	X2310047M10RIK,HIPK2,LEF1,MYB,PDCD1,SOCS1,SOCS3,TCEB2,VHL,WSB1
89	46	6.571	7	CXCR3,PARD3,PRKCI,PVRL1,PVRL3,RAB4A,SMURF1
107	46	6.571	7	CEBPA,CNOT8,JDP2,RB1,RBL1,TBX21,TGFB1I1
67	26	6.5	4	FOXO1,LRPPRC,NR1H3,SIRT1
14	26	6.5	4	BMP2,BMP4,BMP7,SOSTDC1
92	32	6.4	5	EGFR,PDGFRA,PDGFRB,PTEN,SLC9A3R2
60	50	6.25	8	ARHGEF1,EVL,FAS,IPO5,LCP2,SYVN1,VCL,WAS
90	30	6	5	BACH1,HDAC7,MITF,PATZ1,PIAS3
58	18	6	3	IL12B,IL12RB1,IRF1
140	48	6	8	CCNG1,CCNG2,E2F1,GSTA4,NR4A3,SWAP70,TRIM32,TRP53
12	24	6	4	ATP6V0C,BIRC2,BIRC3,HTRA2
120	18	6	3	CISH,LEPR,SOCS2
30	22	5.5	4	CSF1,CSF1R,KIT,SRC
51	66	5.5	12	DNMT1,HDAC1,HNRNPAB,IFRD1,MXD1,MYBL2,PIP5K1B,PRKDC,RCOR2,RUNX2,XPO1,ZFP462
68	16	5.333	3	AHR,MAF,SUMO1
59	32	5.333	6	CASP1,CASP3,CASP8,CEBPB,GZMB,IL1B
63	96	5.053	19	APOE,CASK,CNN3,CTTN,HSP90B1,IPO11,KCNMA1,LDHA,MYO5A,NDEL1,NUDC,PRDX2,RAB6B,ROCK2,SH3BP4,TPM1,TPT1,TRF,TUBB5
114	30	5	6	CARD11,HSP90AA1,KSR1,MALT1,SKIL,SMURF2
23	30	5	6	CD8A,HCLS1,LCK,PIK3CG,RAG2,SPAG9
65	28	4.667	6	LMNA,LMNB1,MYH9,SFN,TOP2A,TRIM28
100	14	4.667	3	PRKCSH,PRKCZ,PTPRC
106	18	4.5	4	ACVR2A,NOS2,PAK1,RAC2
80	22	4.4	5	MYO1C,PCDH15,RICTOR,RRN3,VPS35
11	26	4.333	6	ADAM15,BIN1,DNM1,ITPR1,RASD1,SH3GL3
125	20	4	5	CCL2,CLSTN1,IRF9,STAT2,STX12
101	28	4	7	ADAM12,BAG3,GAB1,NEO1,NRP1,PLXNA1,PTK2
66	16	4	4	CXADR,LNX2,NUMB,SERPINB1A
136	20	4	5	ITGB2,OPTN,PELI2,PPP6C,TNFRSF1A
88	16	4	4	CHORDC1,IGBP1,NR3C1,PPP5C
22	16	4	4	CAV1,CD14,TLR3,UNC93B1
3	20	4	5	APAF1,APC,AXIN2,MAPRE1,MUC1
99	26	3.714	7	ATM,EIF2S1,GSK3A,PPP1R9A,PPP1R9B,RHOB,SKP1A
46	22	3.667	6	E2F2,GATA1,GATA3,GFI1B,LMO2,NFYA
111	18	3.6	5	FZD8,LRP6,RYK,SFRP2,WNT5A
55	14	3.5	4	DYNLL2,HSPB1,HSPH1,PAFAH1B3
137	14	3.5	4	CASP2,SPOP,TRAF1,TRAF5
50	14	3.5	4	HCST,IL2RB,KLRK1,TYROBP
54	14	3.5	4	AES,HMGB1,TLE1,ZFP36
95	10	3.333	3	PLA2G4A,PRDX6,SH3PXD2A
35	10	3.333	3	DZIP3,NCOR1,PPARG
103	10	3.333	3	CEACAM1,PSTPIP1,PTPN12
64	10	3.333	3	FLT1,LDLR,LRP1
78	6	3	2	GMNN,MSX1
49	6	3	2	GRB7,TIA1
115	6	3	2	SLC12A2,STK39
113	12	3	4	DAG1,LAMP1,SGCB,SGCD
123	12	3	4	HDAC10,IVNS1ABP,SREBF2,TFDP2
39	12	3	4	CEBPG,EP300,KAT2A,NUPR1
141	6	3	2	STX7,VAMP8
130	6	3	2	CCT5,TCP1
143	12	3	4	APBB1IP,DIAP1,PFN2,VASP
61	14	2.8	5	CLDN11,CNOT6L,ITGA5,ITGB1,SPARC
112	16	2.667	6	ANXA2,BMYC,GAB2,PRKCE,PRMT5,S100A10
69	8	2.667	3	MAGED1,NDN,ZFP263
145	8	2.667	3	IL6,TNF,XBP1
104	8	2.667	3	ARRB1,BGN,PTS
96	8	2.667	3	PLCB2,POLA1,VIM
47	8	2.667	3	ATXN1,ETS1,GFI1
41	8	2.667	3	BRCA2,CEBPD,FANCD2
70	8	2.667	3	ADRB2,DLL1,MAGI3
40	8	2.667	3	EPAS1,HIF1A,HLF
75	8	2.667	3	MEOX1,MEOX2,TLE4
26	18	2.571	7	CFLAR,EIF4EBP1,FMR1,IRAK2,ITCH,JUNB,STAT1
15	10	2.5	4	BMPR2,FGFR1,MAP3K8,PRKCB
83	10	2.5	4	LITAF,NDFIP1,NEDD4,TDGF1
105	10	2.5	4	MYO7A,RAB27A,RAB3D,SYTL2
2	12	2.4	5	AP2A2,CAPN2,EPS15,GGA2,ITSN1
34	12	2.4	5	DAB2,DTNB,DTNBP1,HAP1,KIF5B
20	6	2	3	CCNT1,EIF2B1,MYC
73	6	2	3	MEF2A,MEF2C,UFC1
121	6	2	3	EGLN3,SPRY2,SPRY4
8	6	2	3	B2M,CD1D1,H2.D1
98	4	2	2	POT1A,TERF1
5	4	2	2	ARL6,ARL6IP5
119	6	2	3	CREM,PRPF40A,SMC2
48	6	2	3	GRB10,IGF1R,MAP3K5
25	8	2	4	CCDC64,CEP290,IQCB1,KIF3A
53	4	2	2	HDAC4,NCF4
42	8	2	4	FBXW7,NOTCH1,NPM1,STAT4
28	6	1.5	4	CLIP1,CLIP2,MAP2K1,NTN1
77	4	1.333	3	CXCL1,MMP12,PAXIP1
81	4	1	4	DDX60,EGR2,NAB2,TRIP13
86	0	0	3	EZR,NGFRAP1,NTF3

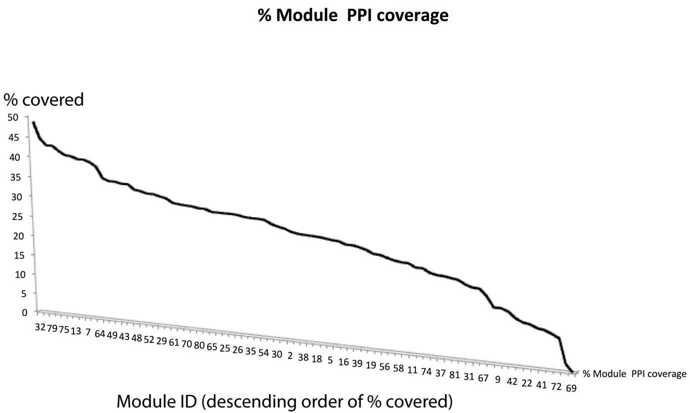
% Module PPI coverage
% covered
50
45
40
35
30
25
20
15
10
5
0
32 79 75 13 7 64 49 43 48 52 29 61 70 80 65 25 26 35 54 30 2 38 18 5 16 39 19 56 58 11 74 37 81 31 67 9 42 22 41 72 69
% Module PPI coverage
Module ID (descending order of % covered)

Suppl Fig. 1
